# Branching Pattern of Inferior Mesenteric Artery in a Black African Population: A Dissection Study

**DOI:** 10.5402/2013/962904

**Published:** 2012-12-24

**Authors:** Simeon Sinkeet, Philip Mwachaka, Johnstone Muthoka, Hassan Saidi

**Affiliations:** Department of Human Anatomy, University of Nairobi, P.O. Box 30197, Nairobi 00100, Kenya

## Abstract

*Background*. Branching pattern of inferior mesenteric artery (IMA) and pattern of vascular supply to the left colon and rectosigmoid areas, though important during colorectal surgery, display interethnic variations. Further, these regions have notable vascular “weak points” reported to be highly susceptible to ischemic colitis. This study aimed at evaluating the branching pattern of IMA in a black African population. *Materials and Methods*. Fifty-seven formalin-embalmed cadavers (28 Male, 27 Female) were studied. The length, branching pattern, and diameter of IMA at its origin were recorded. *Results*. IMA mean length and diameter at origin were 30.57 ± 10.0 mm and 4.10 ± 0.9 mm, respectively. IMA most frequently branched into left colic artery and a common sigmoid trunk in 23 cases while the classical branching pattern was observed in only 7 cases. Colic marginal artery was absent at the splenic flexure and sigmoid colon in 7 and 5 cases, respectively. Arc of Riolan was observed in 9 cases. *Conclusion*. Branching pattern of IMA shows variations from the previously reported cases which might help account for some of the untoward outcomes observed following colon surgery. An angiographic study to further delineate functionality of the arterial anastomoses in this region is recommended.

## 1. Introduction

Arterial supply to the left colon and rectosigmoid colon is from the inferior mesenteric artery (IMA) classically by means of left colic artery (LCA), 2-3 sigmoid branches, and superior rectal artery. IMA originates from the front of the abdominal aorta, near its left margin just below the third part of the duodenum at the level of 3rd lumbar vertebra. It runs downwards arching slightly to the left, and as it crosses the left common iliac, its name arbitrarily changes to the superior rectal (or hemorrhoidal) artery. Pattern of branching however is reported to deviate from this classical description [1–5]. Detailed knowledge of the anatomical variations of the visceral branches of the abdominal aorta is of extreme clinical importance, particularly, when performing laparoscopic abdominal surgery.

Further, colonic vascular supply has some weak areas which have been reported to be highly predisposed to ischemic colitis [6–8]. Griffiths' point at the left colic flexure [9, 10] and the Sudeck's point at the rectosigmoid region [4, 11, 12] are notable examples. In the presence of stenotic or occlusive disease of IMA or superior mesenteric artery (SMA), the presence of collateral channels between either artery is critical for maintaining the integrity of vascular supply to the affected region. Patency and continuity of these collateral channels, namely, colic marginal artery and meandering mesenteric artery, are highly variable. Moreover, knowledge of the pattern of collateral supply also constitutes a critical point of consideration during colic resections in cases of cancer management. The aim of the present study therefore was to examine the variant pattern of blood supply to the left colon and rectosigmoid regions in a black Kenyan population.

## 2. Materials and Methods

The study was carried out using fifty-seven formalin embalmed cadavers (28 Male, 27 Female) obtained from the Department of Human Anatomy, University of Nairobi, used during routine dissection. Any specimen with demonstrable pathology or evidence of previous surgical intervention in the area of interest was excluded from the study. A midline abdominal incision was made. Small intestines were displaced to the right and parietal peritoneum covering retroperitoneal structures on posterior abdominal wall cleared to further reveal the mesenteric arteries and their branches. Inferior mesenteric artery was identified and its vertebral level of origin was determined. Using a sliding vernier caliper SOMET CN-25 1234 (accurate to 1 mm), diameter of IMA at its origin and length of the artery to its point of division were measured. Inferior mesenteric artery branches were identified and followed carefully to the gut wall.

## 3. Results

### 3.1. Vertebral Level of Origin, Length, and Diameter of IMA

Inferior mesenteric artery most frequently gave off the abdominal aorta at the level of the 3rd lumbar vertebra (L3) (38%) being distributed above L3 as far as L1 (31%) and below it up to L4/L5 junction in 31% of the cases. In 91.4% of the cases, it originated between L2 and L4. The origin of the IMA was 6.09 ± 1.46 cm (1.2–8.4 cm) below origin of SMA. Mean length of IMA was 3.56 ± 1.03 cm (range: 2–7 cm) while the mean diameter at the origin was 4.1 ± 0.94 mm (2–6 mm).

### 3.2. Branching Pattern

The most common branching pattern, 23 (39.6%) cases, of inferior mesenteric artery was into LCA and common sigmoid trunk which gave off two (2) sigmoid arteries. Classical branching pattern of IMA was observed in only 7 (12%) cases. Left colic artery most frequently divided into ascending and descending branches in 32 (55.5%) cases.

Sigmoid colon was 26.2 ± 9.5 cm (10.6–51 cm) long and was supplied by 1-2 branches given off by inferior mesenteric artery in 72.4% of the cases. First and second sigmoid branches were both most frequently given off at the level of 4th lumbar vertebra (L4), whereas the third and fourth were given off at L5. Rectosigmoid artery was present in 14 (24.1%) specimens studied.

**Figure 1:**
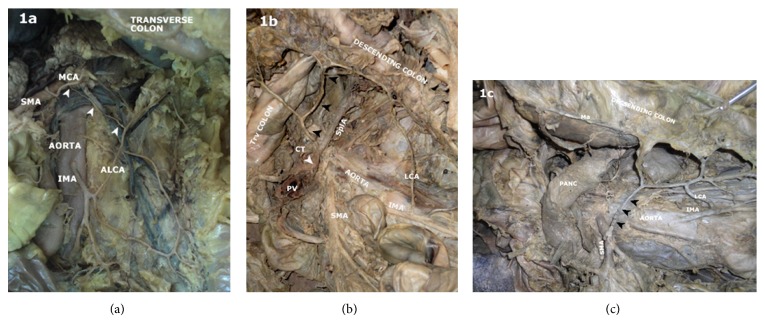
Photograph of the posterior abdominal wall showing the aorta and inferior mesenteric artery (IMA) and its branches. (a) A thin arterial channel (arrowheads) connecting middle colic (MCA) branch of superior mesenteric artery (SMA) to the ascending branch of left colic artery (ALCA) is observed. (b) Arrowheads show an arterial connection between the celiac artery (CT) and the left colic artery (LCA) ascending branch. SplA: splenic artery, PV: portal vein, Trv colon: transverse colon. (c) An inner arterial channel (arrowheads) besides the marginal artery (Ma) connecting the proximal SMA to LCA. PANC: pancreas.

**Figure 2:**
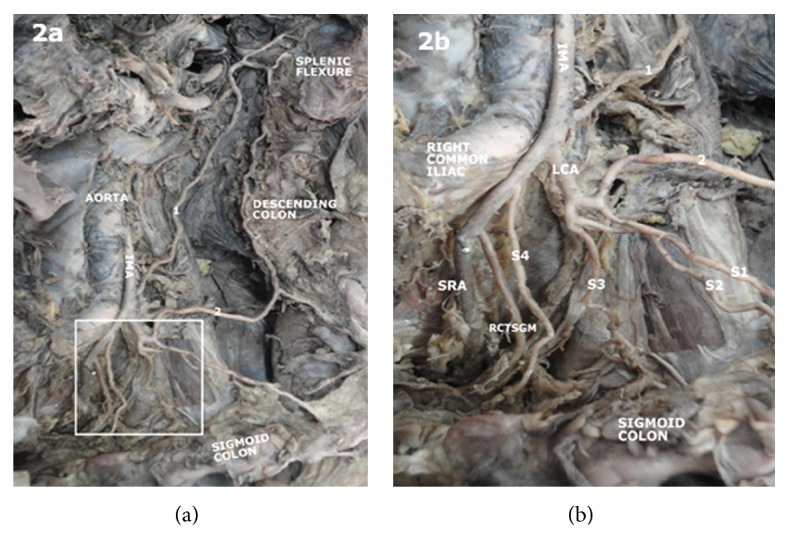
Photograph of the posterior abdominal wall showing the aorta and inferior mesenteric artery as well as its branches (a). The insert is shown in (b). Inferior mesenteric artery (IMA) gave off a left colic artery (LCA) which gave off a transverse branch to the descending colon and proceeded to give three sigmoid arteries (S1, S2, S3) it then proceeds to give a rectosigmoid branch (RCTSGM) and superior rectal artery (SRA).

Some variations, however, deserve a special mention. Connecting arterial channels between left colic artery and superior mesenteric artery (Figure 1(a)), and between left colic artery and celiac artery (Figure 1(b)) was observed. In another case, left colic artery gave off a transverse branch to the descending colon and proceeded to give three of the four sigmoid arteries (Figures 2(a) and 2(b)).

### 3.3. Anastomotic and Collateral Patterns

Macroscopic anastomotic channels from the ascending branch of left colic artery to descending and transverse branches were present in 17 (29.3%) and 7 (12.1%) specimens, respectively. Ascending branch bifurcated into right and left branches in 44 (76%) specimens whereby the distance from point of bifurcation to the gut wall was between 0.2–10 cm (3.37 ± 2.13 cm). Ascending branch of left colic artery assumed a transverse course in 8 (14%) cases. Regarding point of contact with gut wall, this vessel coursed to mid descending colon and splenic flexure in 13 (22.4%) and 44 (76%) cases, respectively. The ascending branch of LCA anastomosed with the left branch of middle colic artery in 54 (93%) specimens was studied.

Colic marginal artery was continuous (external diameter >1 mm), somewhat continuous (<1 mm), and absent at splenic flexure and the sigmoid colon as shown in Table 1. Macroscopic anastomosis between rectosigmoid and inferior sigmoid and superior rectal arteries was seen in 12 (80%) and 11 (73.3%) of the cases. Arc of Riolan, a short part of the colic marginal artery located in the region of the left colic flexure, was observed in 9 (16%) of the specimens (Figure 1(c)).

## 4. Discussion

Inferior mesenteric artery and its branches have been grafted and used as alternatives to the internal thoracic artery in coronary revascularization due to less atherosclerotic involvement and higher success rates [13–15]. In addition, the length of IMA from its origin to the Sudeck's point is critical during ligation of the artery in colorectal surgery [16]. An adequately long IMA therefore will provide for feasibility and safety of such surgical procedures. In the present study, the mean length of IMA was 3.65 ± 1.03 mm which lies within the reported range of 2–7 cm [4].

Classical branching pattern of IMA was seen only in seven (12%) cases which compares with that by Golligher [17] of 15% in a series of 100 cadavers but slightly lower than that reported by Basmajian [16] in 28.9%. Most frequent branching patterns of IMA were left colic artery and a common sigmoid trunk. Concordantly, a common trunk for left colic and sigmoid arteries has been observed in 53–56% of specimens in other populations [4, 9]. Two or three sigmoid arteries have been observed in 96–73% [4, 16, 18] similar to the findings in the present study of 72.4%.

Ascending branch of the left colic artery coursed to splenic flexure in 44 (76%) and to mid descending colon in only 13 (22.4%) cases. This finding affirms the assertion by Lorenzini et al. [19] that vascular continuity of the left colon is not constant enough to assure the surgeon for possible interruptions, whereas splenic flexure on the other hand is a well-vascularised region. Smaller retroperitoneal branches have also been shown to augment the left colic and middle colic arteries in supplying the splenic flexure [19]. This study therefore further supports the need for extra caution during operations in the left colon due to its tenuous blood supply.

Similarly, division of the superior rectal artery distal to the Sudeck's point in the presence of insufficient or lacking anastomosis between the superior rectal artery and the last sigmoid branch has been reported to cause postoperative ischemic stricture [10]. Colorectal surgeons and other surgeons working in this region should therefore always take into consideration this fact to avoid any inadvertent assault to this precarious blood supply.

Colic marginal artery, a tier of the right, middle, and left colic arteries, is usually of small caliber which increases with occlusion of either the SMA or IMA. Meandering mesenteric artery is an arterial trunk connecting SMA and IMA at their roots forming a second colic arterial arcade [20]. Presence of this other collateral channel signifies severe stenotic disease or occlusion of either the SMA or IMA. This arterial channel courses along the base of the colonic mesentery. Running between the roots of the superior and inferior mesenteric arteries, the meandering mesenteric artery unlike the Arc of Riolan is not part of the marginal colic artery [20]. Its absence in this study is concordant with previous reports of a range between 0 and 18% [21, 22].

## 5. Conclusion

Branching pattern of IMA shows variations from the previously reported cases which might help to account for some of the untoward outcomes observed following colon surgery. Careful attempt to delineate the branching pattern of the inferior mesenteric intraoperatively is recommended to avert possible dismal surgical outcome. An angiographic study to further delineate functionality of the arterial anastomoses in this region is recommended in living subjects.

## Figures and Tables

**Table 1:** Continuity of colic marginal artery at splenic flexure and sigmoid colon.

Region	Continuous *n* (%)	Somewhat continuous *n* (%)	Absent *n* (%)
Splenic flexure	45 (77.6%)	5 (8.6%)	7 (12.1%)
Sigmoid colon	39 (68.4%)	13 (22.8%)	5 (8.8%)
